# Attentional capture by real and illusory faces: a failure to replicate

**DOI:** 10.1007/s00426-025-02211-3

**Published:** 2025-11-28

**Authors:** Francesca Miti, Angela Ciaramidaro, Sandro Rubichi, Cristina Iani

**Affiliations:** 1 Department of Biomedical, Metabolic and Neural Sciences, University of Modena and Reggio Emilia, Reggio Emilia, Italy; 2 Center of Neuroscience and Neurotechnology, University of Modena and Reggio Emilia, Reggio Emilia, Italy; 3 Department of Surgery, Medicine, Dentistry and Morphological Sciences with interest in Transplant, Oncology and Regenerative Medicine, University of Modena and Reggio Emilia, Reggio Emilia, Italy

**Keywords:** Attention capture, Visual search, Face effect, Pareidolia

## Abstract

Evidence indicates that faces are rapidly detected and prioritized in visual processing due to their social relevance. Crucially, research has shown that faces capture attention even when they are task-irrelevant, suggesting the existence of an automatic, domain-specific attentional mechanism. This prioritization appears to extend to face pareidolia—the illusory perception of faces in objects—indicating that face detection may rely on a broadly tuned mechanism that classifies stimuli as faces or non-faces based on relatively minimal information, such as the T-shaped configuration common to all faces. To explore whether such a configuration is sufficient to automatically capture attention, we conducted three behavioral experiments using a visual search paradigm. In Experiments 1A (online) and 1B (lab-based), participants searched for a butterfly target while either a real or an illusory face appeared among object distractors. Contrary to expectations, neither real nor illusory faces captured attention. In Experiment 2, in different blocks, participants were asked to search for a real face, an illusory face, or a butterfly presented among object distractors. The results showed that real faces were detected more efficiently than illusory faces or butterfly targets. Crucially, illusory faces showed a search disadvantage even compared to butterflies. Overall, these findings challenge the assumption that a minimal face configuration automatically captures attention. More importantly, these findings challenge the notion that attentional capture by irrelevant faces is entirely automatic, suggesting instead that such capture may be influenced by contextual factors or task demands.

**Supplementary Information:**

The online version contains supplementary material available at 10.1007/s00426-025-02211-3.

## Introduction

Faces convey a wealth of information related to gender, race, and identity, as well as emotional states and the direction of attention, making them highly relevant for social interactions. For a face to be consciously recognized in its expression, identity, or any other feature, a “face element” must first be detected in the environment (Hershler & Hochstein, 2005; Taubert et al., 2017; Tsao & Livingstone, 2008). Studies indicate that face detection is a rapid process, typically taking less than 100 ms (Crouzet et al., 2010), and distinct from facial recognition, that is, the capacity to recognize whether a face has been already seen and to identify the identity (Hershler et al., 2010; Robertson et al., 2017; Tsao & Livingstone, 2008). Indeed, despite their inability to recognize familiar faces, individuals affected by prosopagnosia after brain damage can still detect the presence of a face. Similarly, individuals with developmental prosopagnosia have been shown to perform well in face detection tasks (e.g., Johnson, 2005; Garrido et al.,  2008; see Corrow et al., 2016 for a review).

Neuroimaging studies have revealed that in both humans and monkeys, face processing engages a complex network, encompassing both subcortical regions, such as the superior colliculus, pulvinar, and amygdala, and cortical areas, such as the Fusiform Face Area located in the inferior temporal cortex, that show a preferential response to faces compared to other non-face stimuli (Johnson et al., 2015; Kanwisher & Yovel, 2006; see Kobylkov & Vallortigara, 2024 for a review). While the cortical network supports individual face recognition, the subcortical pathway, that operates outside of conscious awareness, is thought to transmit information to the amygdala through the superior colliculus and pulvinar, enabling the rapid detection of faces and determining the orientation response to face stimuli (e.g., Johnson, 2005; Taubert et al., 2018).

It has been proposed that detection acts as a domain-specific gating stage of processing that filters inputs, and thus permits the prioritized allocation of attentional resources for the subsequent stages of face processing (Tsao & Livingstone, 2008). Support for the idea that faces have prioritized access to attention as compared to other stimuli comes from behavioral studies using different experimental paradigms (e.g., Hershler et al., 2010; Lewis & Edmonds, 2003). For instance, by using the visual search paradigm (Treisman & Gelade, 1980), that has been originally designed to investigate the detection of a target in the presence of non-target elements, it has been shown that the detection of a target face is faster and more efficient compared to the search of other kinds of stimuli (e.g., Hershler & Hochstein, 2005, see also Collyer, Ireland, & Susili, 2024; Crouzet et al., 2010; Hershler et al., 2010). Crucially, using an irrelevant face visual search paradigm, Langton et al. (2008) found that participants took more time to report whether a butterfly (the target) was present or not when a face was presented among other objects (i.e., the distractors) than when it was absent. These results show that faces capture attention even when they are task irrelevant (Langton et al., 2008; Riby et al., 2012) suggesting that attention allocation to faces is an automatic and mandatory process. Such a conclusion seems to be supported also by the results of studies showing that faces are less prone to inattentional and change blindness than objects (e.g., Devue, Laloyoux, Feyers, Theeuwes, & Bredart, 2009; Ro & Lavie, 2001) and that face distractors disrupt target detection in an attentional blink task, relative to other abruptly onsetting distractors (Sato & Kawahara, 2015).

Notably, the sensitivity of the visual system to faces seems to be at the basis of a phenomenon called face pareidolia, that is, the illusory perception of a face in inanimate objects such as clouds, vegetables or houses (Liu et al., 2014; Palmer & Clifford, 2020; Partos et al., 2016; Wardle, Seymour, & Taubert., 2017; Zhou & Meng, 2020). It has indeed been proposed that face detection relies on a broadly tuned mechanism that classifies a stimulus as a face or a non-face based on relatively minimal information, that is, the presence of two eyes, a nose and mouth, that is the T-shaped configuration that is common to all faces (e.g., Itier et al., 2011; Omer et al., 2019). Since illusory faces^1^ share this minimal information, they are thought to activate the general face template, leading to the erroneous perception of a face when there is none (Zhou & Meng, 2020). These false alarms seem to be an adaptive tendency (Akdeniz et al., 2018; Akechi, Kibuchi, Tojo, Osanai, & Hasegawa, 2014; Taubert et al., 2017) that prevents or limits the possibility of missing relevant faces present in the environment (Palmer & Clifford, 2020; Taubert et al., 2017). This phenomenon is also experienced by non-human primates (e.g., Saurels et al., 2024; Taubert et al., 2017) and has been shown to engage neural mechanisms like those engaged by human faces (e.g., Taubert et al., 2022).

Most importantly, there are indications that, similarly to real faces, illusory faces receive prioritized attention as compared to objects (e.g., Jakobsen et al., 2023) and privileged access to conscious awareness (e.g., Caruana & Seymour, 2022). By using pareidolic images in a visual search experiment, Keys et al. (2021) found a clear advantage in locating face-like objects (i.e., illusory faces) compared to matched standard objects. Specifically, in two experiments participants were required to search for a target, cued for 1600 ms at the beginning of each trial, in search arrays of variable set sizes. In Experiment 1, participants were presented with search arrays of 16, 32 or 64 images, displayed in random positions on an invisible search grid. The target could either be an illusory face or a matched object (that is, an example of the same object as the illusory face, but without a face). Matched objects not used as targets were used as distractors. Results showed a search advantage for illusory faces compared to matched objects, irrespective of set size. In Experiment 2, participants were presented with circular search arrays of 4, 8 or 16 images. The target could be an illusory face, a category-matched object or a real face and distractors were category diverse and did not match the target category. Again, illusory faces showed a search advantage compared to matched objects, irrespective of set size. However, search times were significantly faster for real faces as compared to both illusory and matched objects. More recently, in three experiments Collyer et al. (2024) compared search for real faces, illusory faces, matched objects and uniform objects (flowers) in search arrays of 16, 25 or 36 items. They found that searching for illusory faces was less accurate and slower than searching for real faces at all set sizes. Illusory faces showed an advantage compared to real objects only when the targets belonged to variable categories (Experiment 1). No search advantage was evident when illusory faces were contrasted to uniform target objects (flowers) (Experiment 2) and, in addition to including uniform target objects, the target was not cued (Experiment 3). In this latter case, a search disadvantage for illusory faces was observed. Altogether, these latter results seem to indicate that the face-like configuration present in illusory faces may not be sufficient to automatically capture attention. A way to test this hypothesis is to assess whether illusory faces capture attention even when irrelevant to the task.

Given these premises, we conducted three experiments using a visual search paradigm. In the first two experiments, we used a modified version of the visual search task used by Langton et al. (2008, Study 1) in which we asked participants to search for a butterfly amongst a circular display of six items. Either a real or an illusory face could appear among the task-irrelevant distractors along with standard objects. The only difference between the two experiments was that Experiment 1A was conducted online, while Experiment 1B was conducted in the laboratory. If the minimal face configuration drives the prioritized detection of faces, we should observe slower reaction times for both irrelevant real and illusory faces. As we did not find evidence of attention capture for either the real faces or the face-like objects, in Experiment 2 we compared search task performance when faces, illusory faces, and butterflies were used as targets to assess whether the absence of an effect could be due to the specific features of the stimuli used in our study or rather to the task-relevance of the face and face-like stimuli.

## Experiment 1A

In Experiment 1A, we used a modified version of visual search task used by Langton et al. (2008, Study 1) in which participants were required to search for a butterfly amongst a circular display of six items. In our version of the task, a real face or an illusory face could appear among the distractors that were standard objects. Since the images of illusory faces were selected from a validated set of pictures taken from the web, each presenting high variability in visual characteristics (e.g., the variability in perspective, orientation, size, resolution, etc.), the set of images of standard objects, selected among common inanimate objects, and of human faces were selected from different databases to match this variability. Moreover, the selected illusory faces rarely showed a neutral expression, and the objects judged to more closely resemble a face configuration were those with a happy emotional expression (see the stimulus selection procedure described below). Consequently, we selected happy expressions for the human face stimuli too. Since there is evidence that people differ widely in face processing abilities (e.g., Yovel et al., 2014), in addition to the visual search task, we administered a standardised face identification task, the Glasgow Face Matching Task (GFMT) (Burton et al., 2010), to examine whether eventual variability in attentional capture by real or illusory faces could be accounted for by individual differences in face identification skills.

Based on the hypothesis that detecting a face-like configuration alone is sufficient to capture attention, we expected to observe slower reaction times not only when a real face was present but also when an illusory face was present among the distractors, compared to a condition where only standard objects were shown along with the target. The absence of any interference effect from illusory faces would suggest that a face-like configuration alone is insufficient to capture attention.

### Methods

#### Participants

The appropriate sample size was calculated a priori with G*Power (effect size f = 0.25, α = 0.05, power = 0.95) (http://www.gpower.hhu.de/). The power calculation recommended a sample size of at least 28 participants. Since the experiment was conducted online, we did not restrict recruitment to this number but, to ensure an appropriate final sample size, we tested all the available participants, which were 41.

Participants were recruited from a pool of students from the University of Modena and Reggio Emilia who previously declared to have an interest in being recruited for psychology studies. The experiment was presented as an online study on visual perception. Five participants were excluded due to technical problems or excessive noise and distraction during the sessions. The final sample included 36 participants (24 females) with a mean age of 28.03 years (SD = 11.5). All of them had normal or corrected-to-normal vision. This and the following experiments were conducted in accordance with the ethical standards laid down in the 1964 Declaration of Helsinki and its later amendments and were approved by the Ethics Committee of the University of Modena and Reggio Emilia (protocol n. UNMRCLE-0026506). All participants gave their informed consent to participate.

Participants were required to perform two different tasks in fixed order: the visual search task and the Glasgow Face Matching Task (GFMT) (Burton et al., 2010).

#### Materials

In the visual search task, three types of stimuli were used as distractors: faces, illusory faces, and standard objects. Eight images of butterflies were used as targets. To select the illusory faces, we conducted an online stimulus pre-selection study to ensure that the images elicited the illusion of a face. For this study, an independent sample of 20 judges (10 female; mean age 27.6 years, SD 8.8 years) rated 180 images of illusory faces. The images were downloaded with the Google search engine and presented using Inquisit Web. While viewing each picture, participants were asked to rate the similarity to a face for each of the 180 objects on a scale ranging from 0 (not similar at all) to 6 (the illusion of a face is unambiguous). For ratings of 1 or higher, participants were asked to indicate the emotion associated with the illusory facial expression, choosing among the 6 basic Ekman emotions (Ekman et al., 1987) or the option “no emotion”.

From the complete set of 180 images, we selected the eight pictures with the highest ratings (equal or above 4.5) of face-likeness, these being objects judged to show a happy facial expression. Eight happy human faces (4 females and 4 males) and fifty-four standard inanimate objects were selected from three different picture databases: IAPS (Lang et al., 1997), Oasis (Kurdi et al., 2017), and EmoMadrid (Carretié, Tapia, López-Martin, & Albert, 2019). All these pictures have been used in many different studies and have been evaluated on different dimensions, allowing us to avoid biases in choosing images that could present specific visual characteristics. After selection, the images were cropped and modified to appear in black and white. The final set of stimuli consisted of 72 images: 8 happy faces, 8 illusory faces with a happy expression, 48 standard objects, and 8 butterflies (real and illusory faces are reported in the Appendix). Eight objects were randomly selected from the set of 48 standard objects to be displayed an equal number of times as the 8 faces and 8 illusory faces, balancing for repetition effects between conditions.

#### Visual search task

**Fig. 1:**
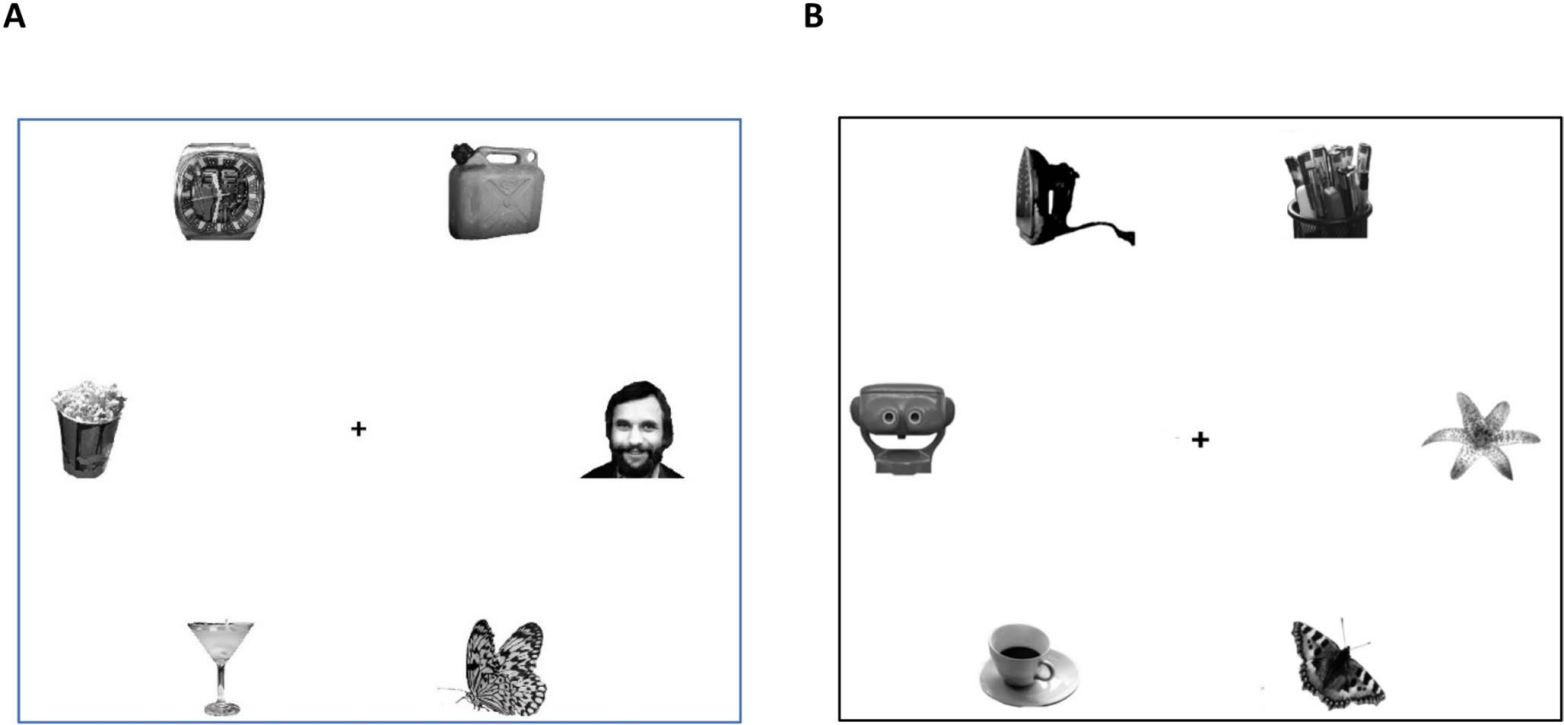
Example of the search display used in Experiments 1A and 1B. The (**A**) panel shows a target-present, face-distractor trial, whereas panel (**B**) shows a target-present, illusory face-distractor trial

The visual search task was built following Langton et al. (2008). The stimuli were sized to fit a 3 × 3 cm square, independently of the size of the monitor, and displayed in a circle at a visual angle of approximately 4.3° from the fixation cross at the center of the white screen (see Fig. 1).

#### Glasgow face matching task (GFMT)

As a face-perception control task, the shortened version of the GFMT was employed (Burton et al., 2010), which consists of 40 images depicting two faces presented side-by-side. Half of the stimulus set depicts two full-face view pictures of the same person taken with different cameras, while the other half shows pictures of two different people. For each pair, participants are asked to decide whether the two images represent the same person or two different people.

#### Procedure

Both tasks (Visual search and GFMT) were programmed using Inquisit 6 (Millisecond Software) and administered online in a single session (for quality-check of data collected using online experiments, see Schubert et al., 2013; Semmelmann & Weigelt, 2017). Participants were contacted through Google Meet and instructed to download the Inquisit Web plug-in on their computer. The experimenter monitored participants via the internet during all tasks without interacting with them unless necessary. This was done to monitor performance, and to check for noise, distractions, and participants’ behavior. We asked participants to carry out the experiment sitting in a comfortable chair in front of their computer, rigorously placed on a table or desk. The monitor size was measured at the beginning of the experiment through an online app (https://www.piliapp.com/actual-size/what-is-my-monitor-size/) and controlled for later in the analyses.

The visual search task was always presented first. Participants were asked to determine as quickly and accurately as possible if a butterfly was present or not in the circle array. No information about images was provided, so neither real nor illusory faces were mentioned before starting the task. Half of the subjects were instructed to press the E or Y key when the target was present and the O or P key when the target was absent. Key specifications depended on the presence or absence of the number pad on the participant’s keyboard (E and O for no numpad). The opposite keys for target present and target absent responses were assigned to the other half of the subjects. Each trial began with a fixation cross with jittered duration (from 100 to 1500 ms) followed by a 6-item circle that remained visible until response. The stimuli disappeared after 2 s if no response was given.

The task consisted of 120 trials per distractor type (face, illusory face, and standard object) divided into 60 target-present trials and 60 target-absent trials, for a total of 360 randomly presented trials. Stimuli selection and position were randomly generated by the program so that each specific image would appear in each one of the six positions with approximately the same frequency. The session consisted of 3 blocks so that participants could rest every 120 trials. Twelve practice trials with error feedback were proposed before the experimental trials.

After completing the visual search task, participants were allowed to rest before performing the GFMT, which consisted of the presentation of the 40 images. Half of the participants were instructed to press the E or the Y key (depending on the presence of a numpad on the keyboard) for the “same-person” judgements and the O or P key for the “different-people” judgements. The opposite keys were assigned to the other half of the sample. The images were presented until a response was given. No time constraints were specified.

## Results

For this and the following experiments, statistical analyses were performed using IBM SPSS Statistical software (version 25.0) and JASP (version 0.95; JASP Team, 2025).

### Visual search task

The error rate was 2.8%. To approach normal distribution and make data more suitable for statistical analyses, error proportions were arcsine transformed. Correct mean reaction times and the arcsine transformed error proportions were submitted to two separate repeated measures analyses of variance (ANOVA) with Trial type (target-present vs. target-absent) and Distractor type (face vs. illusory face vs. standard object) as within-participant factors. The partial eta squared was used as a measure of effect size, and following significant interactions, pairwise comparisons were computed using the Bonferroni correction. The respective means are shown in Tables 1 and 2. For ease of understanding, even though the error analysis was performed on transformed values, in Table 2, we present the error proportions.

**Table 1:** Mean reaction times (and standard error) in milliseconds from Experiment 1A for the three distractors in the target-present and target-absent trials

	Distractor type		
Trial type	Face	Illusory face	Standard object
Target-present	683 (12)	675 (13)	687 (14)
Target-absent	855 (20)	845 (19)	853 (20)

**Table 2:** Mean error proportions (and standard error) from experiment 1A for the three distractors in the target-present and target-absent trials

	Distractor type		
Trial type	Face	Illusory face	Standard object
Target-present	0.034 (0.005)	0.028 (0.004)	0.037 (0.005)
Target-absent	0.018 (0.005)	0.026 (0.005)	0.024 (0.005)

The analysis of RTs showed a significant main effect of Trial type with longer reaction times for target-absent (M = 851 ms, SE = 19) compared to target-present (M = 681, SE = 13) trials, F(1,35) = 257.84, *p* <.001, η_p_^2^ = 0.88. The main effect of Distractor type was also significant, F(2,70) = 3.27, *p* =.04, η_p_^2^ = 0.08. Although RTs were slightly faster when an illusory face appeared among the distractors, pairwise comparisons did not show significant differences between distractors, ps > 0.10. The Trial type x Distractor type interaction was not significant, F < 1.

To quantify the relative evidence for the presence or absence of effects rather than solely relying on null-hypothesis significance testing, we conducted a Bayesian repeated measures ANOVA using JASP with uninformed (default) priors. Bayesian model comparison was performed using Bayes factors expressed as BF₀₁, where each model is compared against the best-fitting model in the set. Thus, BF₀₁ >1 indicates that the best-fitting model is more likely than the model under consideration. For evaluating the contribution of individual predictors across models, we report model-averaged inclusion Bayes factors (BF_incl), where values greater than 1 indicate evidence for including the predictor and values less than 1 indicate evidence for excluding it (Lee & Wagenmakers, 2013). Model comparison indicated that the data were 1.39 times more likely under the Trial type–only model than under the model that additionally included Distractor type, and 11.56 times more likely than under the model that included both main effects and their interaction. Consistently, the inclusion Bayes factor for Trial type was greater than 100, providing extreme evidence for its inclusion, while evidence against inclusion was anecdotal for Distractor type (BF_incl = 0.49) and moderate for the interaction (BF_incl = 0.19).

The analysis of errors showed only a main effect of Trial type, F(1,35) = 8.91, *p* =.005, η_p_^2^ = 0.20, indicating that participants committed more errors during target-present (M = 0.158, SE = 0.011) than during target-absent (M = 0.115, SE = 0.014) trials. Neither the main effect of Distractor type, F(2,70) = 1.03, *p* =.36, η_p_^2^ = 0.03, nor the interaction between Trial type and Distractor type, F (2, 70) = 2.48, *p* =.09, η_p_^2^ = 0.07, was significant.

After completion of the task, we asked participants if they became aware of any human faces or face-like objects in the visual search arrays during the experiment. Thirty-two participants out of 36 (89%) reported noticing the human faces, while only 14 of the participants (39%) reported noticing pareidolic faces and were able to accurately describe at least one face-like object. Control analyses performed excluding the four participants who did not notice real faces and the 22 participants who did not notice illusory faces, confirmed the results of the analysis performed on the data of the entire sample.

### GFMT

For each participant, we calculated the accuracy rate (percentage of correct answers). The performance of the total sample was comparable to normative data (Burton et al., 2010): the mean percentage of correct responses was 80%, with a minimum value of 50% and a maximum of 98%. As we proposed this test as a control for face perception abilities, we set a cut-off point at 0.75 based on normative data and excluded from the analysis of RTs eight participants who scored lower. This analysis confirmed the significant main effect of Trial type, F(1,24) = 174.48, *p* <.001, η_p_^2^ = 0.89, while neither the main effect of Distractor type, F(2,48) = 1.41, *p* >.10, η_p_^2^ = 0.05, nor the interaction between the two factors, F < 1, was significant.

## Discussion

Based on the results of previous visual search studies (e.g., Langton et al., 2008), we expected to find longer reaction times when a face was presented as a distractor compared to when it was absent. Furthermore, if the configuration of a face without all features typical of faces is sufficient to capture attention, we expected longer reaction times also when an illusory face was presented along with the target and the other distractors. None of these predictions were proved true. Indeed, neither RTs nor accuracy differed as a function of distractors, even when we excluded from the analyses participants who showed lower face perception abilities. These results could point to the conclusion that in our task, neither faces nor illusory faces captured attention in a mandatory fashion. However, before drawing such a conclusion, we should exclude the possibility that our results were due to the online administration of the experiment. To this end, we replicated Experiment 1A in the laboratory.

## Experiment 1B

To exclude the possibility that the results observed in Experiment 1A were due to online administration, we replicated the experiment in the laboratory. In addition to asking whether participants had noticed the presence of faces and/or illusory faces among the distractors, we administered a recognition test at the end of the visual search task to have a clear indication of whether these stimuli were noticed and processed.

## Methods

### Participants

Thirty new participants (20 females) with a mean age of 25.7 years (SD = 19.61) were recruited as in Experiment 1A.

### Design, materials, and procedure

Participants performed the same tasks as in Experiment 1A, sitting in front of a computer approximately 60 cm from the screen. Stimuli were the same as those used in Experiment 1A. They subtended a visual angle of 2.86 degrees (image size: 3 × 3 cm) from a viewing distance of 60 cm and were presented in a circle, 4.3 degrees of visual angle from the center of the screen.

Unlike Experiment 1A, a standard keyboard was used and therefore all participants used the same keys (E/O) to respond. As in Experiment 1A, at the end of the visual search task, participants were asked if they had noticed the presence of a face and/or an illusory face. To clarify and elaborate on their response to this question, participants were presented with sets of 8 stimuli from each category used as distractors. All stimuli were shown simultaneously, one category at a time, on a computer screen, alongside with 16 new images from the same category that had not been used as experimental stimuli. The experiment then asked the participants to verbally indicate whether they could recognize any stimuli from the experiment and, if so, which ones. The experimenter recorded the answers.

## Results

### Visual search task

The errors rate was 3.4%. Correct mean reaction times and the arcsine transformed error proportions were analyzed as in Experiment 1A. The respective means are shown in Tables 3 and 4.

**Table 3:** Mean reaction times (and standard error) in milliseconds from experiment 1B for the three distractors in the target-present and target-absent trials

	Distractor type		
Condition	Face	Illusory face	Standard object
Target-present	613 (15)	619 (15)	619 (15)
Target-absent	755 (26)	760 (24)	761 (26)

**Table 4:** Mean error proportion (and standard error) from Experiment 1B for the three distractors in the target-present and target-absent trials

	Distractor type		
Condition	Face	Illusory face	Standard object
Target-present	0.042 (0.018)	0.040 (0.007)	0.045 (0.006)
Target-absent	0.034 (0.063)	0.025 (0.000)	0.019 (0.000)

The analysis of RTs showed a significant main effect of Trial type with longer reaction times for target-absent (M = 758,85 ms, SE = 25) than for target-present (M = 617, SE = 15) trials, F(1,29) = 114.09, *p* <.001, η_p_^2^ = 0.80. Neither the main effect of Distractor type, F(2,58) = 1.70, *p* =.19, η_p_^2^ = 0.05, nor the interaction between the two factors, F < 1, was significant^2^.

As for Experiment 1A, we performed a Bayesian analysis using JASP (JASP Team, 2025). The Bayes factor analysis indicated that the data were best explained by a model including only Trial type. Specifically, the data were 3.91 times more likely under the Trial type-only model than under a model also including Distractor type, and 43.2 times more likely than under a model including both main effects and the Trial type × Distractor type interaction. There was very strong evidence for including Trial type (BF_incl > 100), moderate evidence against including the main effect of Distractor type (BF_incl = 0.17), and strong evidence against including the interaction (BF_incl = 0.08). These results suggest that neither Distractor type nor the interaction contributed meaningfully to explaining the data.

The error analysis revealed only a significant main effect of Trial type, F(1,28) = 18.56, *p* <.001, η_p_^2^ = 0.40, with participants being more prone to errors during target-present (M = 0.184, SE = 0.012) than during target-absent (M = 0.131, SE = 0.014) trials. Neither the main effect of Distractor type, F(2,56) = 1.84, *p* =.17, η_p_^2^ = 0.06, nor the interaction between Trial type and Distractor type, F(2, 56) = 2.51, *p* =.09, η_p_^2^ = 0.08, reached significance.

Twenty-three participants out of 30 (77%) reported noticing human faces, while only 14 participants (47%) reported noticing illusory faces. However, when asked to recognize the distractors used in the experiment, participants reached a mean accuracy of 28% across all distractor categories (i.e., 2.28 out of 8 items per category). Specifically, they correctly identified 25% of the faces, 27% of the illusory faces, and 34% of the standard objects.

### GFMT

The performance of the total sample was comparable to normative data (Burton et al., 2010): the mean percentage of correct responses was 85%, with a minimum value of 57% and a maximum of 100%. As for Experiment 1A, we set a cut-off at 0.75 based on normative data, thus excluding from the RTs analysis 3 participants who scored lower. This new analysis confirmed the main effect of Trial type, F(1,26) = 100.17, *p* <.001, η_p_^2^ = 0.79, while neither the main effect of Distractor type nor the interaction between the two factors was significant, Fs < 1.

## Discussion

As in Experiment 1A, neither real nor illusory faces interfered with the search for the target stimulus. Importantly, the results of the Bayesian analysis provided moderate to strong evidence that neither Distractor type nor the interaction contributed meaningfully to explaining the data. Since the same results were found in the two experiments, we believe that they reliably indicate that, at least in our task, these two types of distractors did not automatically capture attention. Importantly, in both Experiments 1A and 1B, most of the participants noticed faces, but about half of them noticed illusory faces. Moreover, when in Experiment 1B, participants were asked to recognize the stimuli previously presented as distractors, they showed a very low performance with all three distractor types, suggesting that participants allocated minimal attention to all distractors. It should be noted, however, that this post-test may be more suitable for evaluating whether participants retained specific information about these stimuli - which was not necessary for performing the search task - rather than for determining whether participants noticed the irrelevant faces (whether real or illusory). Therefore, the results should be interpreted with caution.

The lack of an interference effect exerted by illusory faces is in line with the results of a recent study by Collyer et al. (2024) showing that even when relevant for the task, illusory faces did not show the same search advantage as real faces. On the contrary, they showed a search advantage over objects only under specific circumstances, that is, when the target was cued, and targets belonged to variable categories. When either uniform object targets (flowers) were used or, as in our Experiments 1A and 1B, targets were not cued, illusory faces showed a search disadvantage compared to both real faces and objects.

More surprising is the lack of an effect of real faces, a result that is in contrast with the results reported by Langton et al. (2008) using a similar paradigm. This discrepancy may be explained by considering the differences between our stimuli and those employed by Langton and colleagues (2008). Specifically, we employed faces and objects that were more heterogeneous and naturalistic. Furthermore, given that there is evidence showing that face emotion and gaze direction may also affect attention deployment (e.g., Ricciardelli et al., 2012; Ricciardelli et al., 2016; Scerrati et al., 2022), most studies investigating face effects used neutral facial expressions. Additionally, faces were oriented frontally - similar to other distractors and targets - and details such as hair were removed (Langton et al., 2008; Pereira Birmingham, & Ristic, 2020; Riby et al., 2012). Differently, in our Experiments 1A and 1B, faces were cropped from preexisting images that presented a happy expression and other confounding elements (such as hair, ears, moustaches, etc.) and were shown in different orientations. Objects and butterfly pictures were taken from different perspectives as well (e.g., three out of eight target butterflies had almost closed wings) and were highly heterogeneous. Indeed, we used inanimate objects belonging to no specific category, while Langton et al. (2008) used plant-related distractors only.

Notably, since the paradigm we implemented was closely modelled on the one used by Langton et al. (2008) who found slower reaction times when an irrelevant face was present and that the only significant variation that we applied to the paradigm was related to the type and characteristics of the stimuli used, our data seem to indicate that the irrelevant face effect may not be as robust as previously suggested.

However, before drawing conclusions from our results, it is necessary to determine whether the real and illusory faces used as distractors in Experiments 1A and 1B show a search advantage when used as targets. Observing such an advantage would suggest that their capacity to capture attention when presented within search arrays such as those used in our study is dependent on the current behavioral goals of the observers. Furthermore, if both real and illusory faces demonstrate a comparable search advantage, this would indicate that the minimal face configuration common to both stimuli is sufficient to influence target processing.

## Experiment 2

### Methods

### Participants

Thirty new participants (24 women) with a mean age of 27.73 years (SD = 4.55 years), recruited as in Experiment 1B, participated in this experiment.

#### Materials, design and procedure

Participants performed a visual search task followed by the GFMT task. For the visual search task, the target varied across blocks and could be either a butterfly, a face, or an illusory face, while the distractors were always standard objects. Differently from Keys et al. (2021) and from Collyer et al. (2024, Experiments 1 and 2), targets were not cued before the array appeared, but participants were told which target type they were searching for before each block. The stimuli were the same as those used in the previous experiments. All other task parameters were identical to the visual search task used in the previous experiments (e.g., keyboard keys, fixation and trial duration, stimuli configuration, etc.). Each block consisted of 120 trials and was preceded by 12 practice trials with error feedback. To account for potential task order effects, the sample was divided into three groups, each experiencing a different order of presentation of the target condition: butterfly, face, illusory face vs. butterfly, illusory face, face vs. illusory face, butterfly, face.^3^

## Results and discussion

### Visual search task

The error rate was 3.6%. Mean correct reaction times and arcsine-transformed error proportions were submitted to two separate repeated measures ANOVAs with two within-participant factors: Trial type (target-present vs. target-absent) and Target identity (butterfly, face, illusory face). When necessary, post-hoc comparisons were performed using the Bonferroni correction. The respective data are shown in Tables 5 and 6.

**Table 5:** Mean reaction times (and standard error) in milliseconds from experiment 2 for the three target identities in the target-present and target-absent trials

	Target identity		
Trial type	Butterfly	Face	Illusory face
Target-present	676 (15)	613 (16)	883 (16)
Target-absent	837 (26)	693 (18)	1154 (27)

**Table 6:** Mean error proportions (and standard error) from experiment 2 for the three target identities in the target-present and target-absent trials

	Target identity		
Trial type	Butterfly	Face	Illusory face
Target-present	0.030 (0.005)	0.024 (0.004)	0.087 (0.010)
Target-absent	0.021 (0.007)	0.011 (0.003)	0.036 (0.004)

The analysis of RTs showed significant main effects of Trial type, F(1, 29) = 161.68, *p* <.001, η_p_^2^ = 0.89, and Target type, F(2,58) = 296.35, *p* <.001, η_p_^2^ = 0.91. RTs were faster in target-present trials (M = 724 ms, SE = 13) as compared to target-absent trials (M = 895 ms, SE = 21) and when the target was a face (M = 653, ms, SE = 16) as compared to when it was either a butterfly (M = 757, ms, SE = 19) or an illusory face (M = 1019, SE = 20). Pairwise comparisons showed that all target types differed from each other (all ps < 0.01). The interaction between Trial type and Target identity was also significant, F(2, 58) = 72.47, *p* <.001, η_p_^2^ = 0.71, and was mainly driven by the difference in RTs between target-present and target-absent trials, being the highest when the target was an illusory face compared to when it was a butterfly or a face.

As for the previous experiments, we performed a Bayesian analysis using JASP (JASP Team, 2025). The Bayes factor analysis indicated that the data were best explained by a model including both main effects of Target type and Trial type, as well as their interaction. Specifically, the data were 6.44 × 10^+ 13^ times more likely under this model than under a model including only the two main effects, 1.31 × 10^+ 24^ times more likely than under a model including only the main effect of Target type, and 4.03 × 10^+ 41^ times more likely than under a model including only the main effect of Trial type.

The analysis of errors revealed significant main effects of Trial type, F(1,29) = 70.82, *p* <.001, η_p_^2^ = 0.71, and Target identity, F(2,58) = 24.128, *p* <.001, η_p_^2^ = 0.45. Participants were more likely to miss present targets (M = 0.190, SE = 0.012) than reporting absent targets (M = 0.106, SE = 0.012) and were less accurate when the target was an illusory face. There was also a significant interaction between the two factors, F(2,58) = 4.578, *p* = 014, η_p_^2^ = 0.14. Post-hoc comparisons confirmed that this interaction was mostly driven by the difference in accuracy between target-present and target-absent trials being the highest when the target was an illusory face.

### GFMT

The performance of the total sample was comparable to normative data (Burton et al., 2010): the mean percentage of correct responses was 85%, with a minimum value of 63% and a maximum of 100%. As in the previous experiments, we set a cut-off at 0.75 based on normative data, thus excluding from the RT analysis 6 participants that scored lower. This new analysis confirmed the results of the analysis performed on the entire sample, that is, the significant main effects of Trial type, F(1,23) = 111.51, *p* <.001, η_p_^2^ = 0.202, and Target identity, F(1,23) = 190.15, *p* <.001, η_p_^2^ = 0.62, and a significant interaction between the two factors, F(1,23) = 54.79, *p* <.001, η_p_^2^ = 0.05.

In line with the existing literature, we found that the search for real faces was faster and more accurate compared to illusory faces and butterflies. On the contrary, illusory faces displayed a search disadvantage in both RTs and accuracy, hence indicating that a face-like configuration alone is not sufficient to ensure prioritized access to attention.

#### General Discussion

The main objective of the present study was to assess whether irrelevant illusory faces capture attention in a visual search task. In two experiments, we adapted the visual search task used by Langton et al. (2008, Study 1) and required participants to search for a butterfly in an array containing a total of six items. A real face or an illusory face could appear among the distractors that were standard objects. In neither of the two experiments did we find evidence of attention capture by illusory faces and, even more strikingly, by real faces. Given these results, in a third experiment, we assessed whether real and illusory faces showed a visual search advantage compared to butterflies when relevant to the task. Crucially, we found a search advantage for real faces, whereas a search disadvantage was evident for illusory faces.

Two conclusions can be drawn from these results. First, the presence of a search disadvantage for illusory faces, along with the absence of the irrelevant illusory face effect, suggests that the minimal face configuration common to illusory faces may not be sufficient to elicit privileged attentional biases. To note, such a conclusion conflicts with the results by Keys et al. (2021), who found faster search times for illusory faces compared to matched objects when the distractors were either matched objects (Experiment 1) or variable objects (Experiment 2). However, it is important to highlight that in the study by Keys et al. (2021), the search advantage for illusory faces emerged only when these stimuli were compared to objects but not when they were compared to real faces. This latter finding suggests that, even though illusory faces seem to be processed quickly, their search advantage is not comparable to that shown by real faces. Furthermore, the target was always cued. Recently, Collyer et al. (2024) found that illusory faces showed a search advantage over objects when the target was cued, and targets belonged to variable categories. This advantage was, however, smaller than that shown by real faces and turned to a disadvantage when the variable object targets were replaced with uniform object targets (flowers) and the targets were not cued before each trial. These findings were interpreted to support the view that real faces are searched using highly efficient processes, resulting in their “pop-out” among highly variable distractors, and to suggest that the sensitivity of the visual system to real faces is likely to be guided by the low-level visual characteristics that distinguish them from other stimuli, such as the amplitude spectrum, spatial frequency, and contrast patterns. Unlike real faces, illusory faces show a greater variability at the image level, and since they share some features also with objects, they may compete with them for attention [see Supplementary Material for the results of the analyses assessing differences in image characteristics among the different stimulus categories used in the present study]. This similarity may render distractor rejection less efficient, hence slowing down search times (Horstmann et al., 2006). Alternatively, it is possible that the slower performance with illusory faces was due to their location on the screen. Specifically, our stimuli were presented at 4.3° of visual angle from the centre of the screen. Given a viewing distance of 60 cm, foveal vision encompasses an area of approximately 5° of visual angle, meaning that only part of the stimuli fell within the foveal region. Thus, it is possible that detection of illusory faces, due to their physical characteristics, was more impaired compared to both real faces and butterflies. Since we did not assess observers’ ability to categorize illusory faces when presented in isolation, we cannot conclusively exclude this explanation. However, it should be noted that previous studies reporting a search advantage for illusory faces over standard objects used stimuli of similar or even smaller size, presented at comparable or greater eccentricities (e.g., Keys et al., 2021). Furthermore, a recent study by Saurels et al. (2024) showed that the face pareidolia illusion is independent of foveal vision and survives in peripheral vision (10.8° from central fixation).

Second, we believe that our results extend these previous findings by suggesting that the irrelevant face effect may be less robust than originally thought. More precisely, even though we found a reliable search advantage for relevant faces, we did not find an effect of the irrelevant real faces. It is possible that our results were driven by the specific features of the stimuli used in our study. Indeed, we employed faces and objects that were more heterogeneous and visually more complex compared to those used in previous studies, thereby enhancing their ecological validity, an aspect particularly relevant given prior evidence of attentional capture by irrelevant faces (e.g., Langton et al., 2008).

Hence, one may argue that differences in the featural complexity of the stimuli (targets and face or non-face distractor pictures) may have influenced the strategies adopted by the participants in our task. In other words, one could speculate that in our study, neither illusory nor real faces pop out because of the featural complexity of all the stimuli present in the array, which increased the perceptual load of the task. Such a view is in line with Forster and Lavie (2021), who showed that irrelevant distractors do not interfere with the processing of relevant targets when task load is high (see also Forster & Lavie, 2008a, b). Similarly, Palermo and Rhodes (2007) showed that faces do not pop out when presented among stimuli matched on low-level features, a result suggesting that attentional resources are needed to detect a facial configuration. Therefore, it is possible that the stimuli used in Experiments 1A and 1B were resource-consuming enough to leave no spare capacity for processing irrelevant faces. This finding aligns with the results of studies using distracting faces in paradigms other than visual search showing that even though they might be processed to some extent, as supported by the analysis of Event Related Potentials (ERP), irrelevant faces did not slow down behavioral responses under high load conditions during an irrelevant distractor paradigm (Neumann, Vidka, van Huis, & Palermo, 2018) or a gender-symbol classification task (Hauthal et al., 2012). We cannot neglect that there are indications that faces are still processed under load (e.g., Lavie, Ro, & Russel, 2003; Ro et al., 2007). As suggested by Forster and Lavie (2021), however, it may be possible to reconcile this apparent inconsistency in the literature. Specifically, in the studies by Lavie et al. (2003) and Ro et al. (2007), the face distractors could be relevant to some of the attentional settings employed in the task. By contrast, studies that have reported load-dependent modulations of attentional capture by faces—including our own—employed face stimuli that were completely irrelevant to the task at hand. Notably, our findings align with the increasing converging evidence suggesting that attentional capture by irrelevant faces may be task and context mediated (e.g. Pereira et al., 2020). Specifically, it has been reported that irrelevant faces did not capture attention more than other distractors during a Stroop task (Henschel et al., 2021). Furthermore, when asked to make a saccade to a color singleton while photos of neutral or angry faces, which were irrelevant to the task, appeared among other objects within the search array, participants were able to successfully maintain goal-relevant oculomotor behaviour on most trials, thus resisting reflexive orienting (Devue & Grimshaw, 2017). Importantly, this study found greater oculomotor capture by neutral upright faces (i.e., more canonical faces) than by angry faces or butterflies. This finding may help explain why faces did not capture attention in our study. Indeed, the faces used in our experiments were less canonical, as they displayed a happy expression and were taken from different orientations.

Recently, Qiu, Becker, and Pegna (2023) used an inattentional blindness paradigm and electroencephalography to examine whether faces captured attention under different conditions of task relevancy and awareness. They found that the N2-posterior-contralateral (N2pc) component, considered an electrophysiological marker for attention capture (e.g., Eimer & Kiss, 2007), was elicited by face stimuli only when participants were aware of the faces and when faces were relevant to the task. This component was not observed when participants were unaware of the presence of faces or when faces were irrelevant to the task.

To conclude, the results of the present study showed no evidence that illusory or real faces that are irrelevant to the task capture attention. However, when relevant to the task, the real faces demonstrated a search advantage, while the illusory faces exhibited a search disadvantage compared to the real faces and the butterflies. These findings underscore significant differences between real and illusory faces, suggesting that the attentional capture by real faces may not be as automatic as previously assumed. Instead, this effect appears to be influenced by specific stimulus characteristics and task-related goals. Further research is required to clarify how these factors interact to modulate the extent to which faces capture attention, offering new insights into the mechanisms underlying social perception.

## Appendix

2

**Fig. 2:**
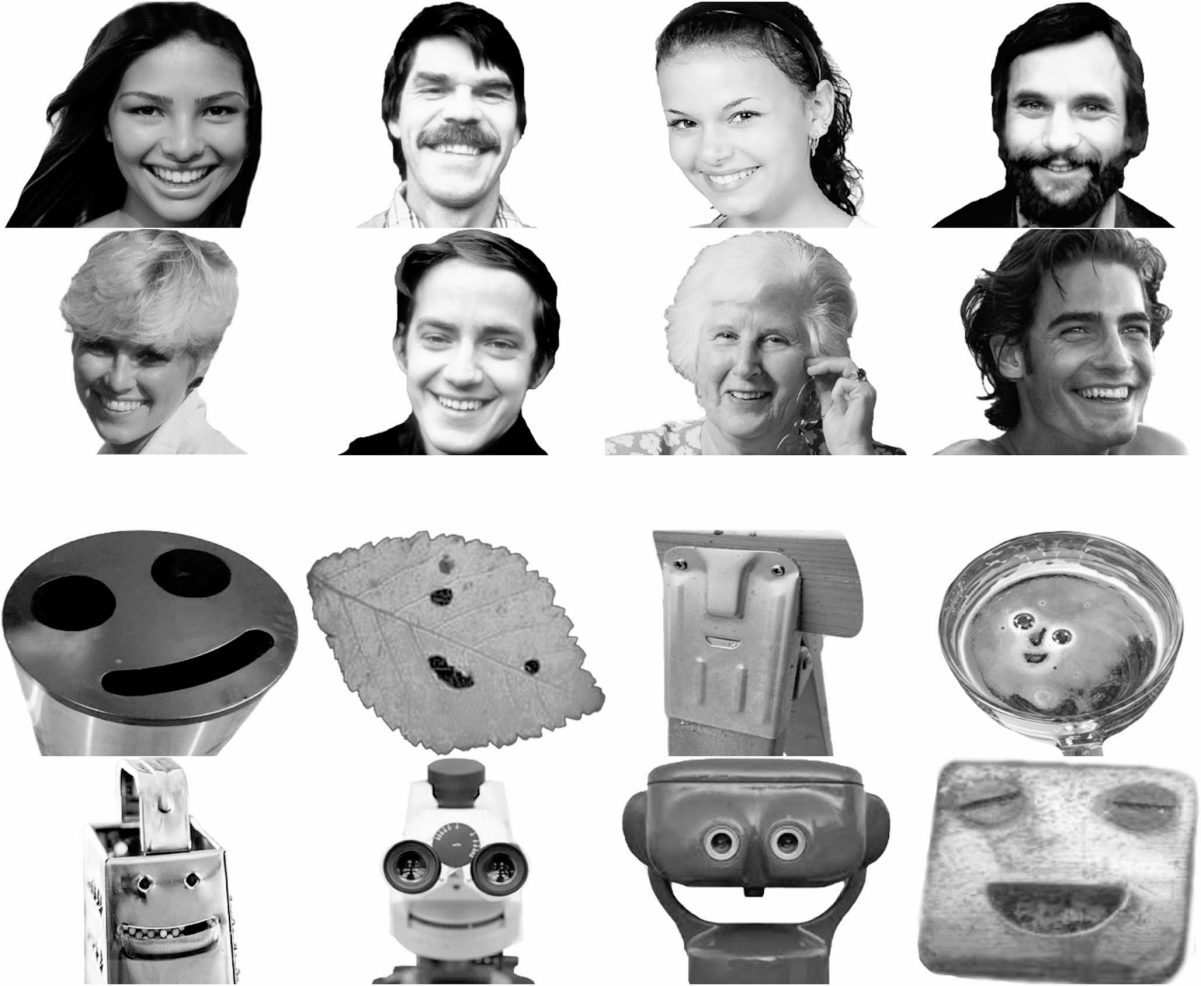
Real andillusory faces used in the study

## Supplementary Information

Below is the link to the electronic supplementary material.

Supplementary Material 1 (DOCX 3.49 MB)

## Data Availability

The dataset analyzed during the current study is available upon request from the corresponding author.

## Footnotes

1 In the different studies, the terms “pareidolic faces”, “illusory faces” and “object-like faces” are used interchangeably to refer to inanimate objects spontaneously perceived as faces. In the manuscript, we will use the term “illusory faces” to refer to these stimuli.

2 To examine whether distractors positioned near the target captured attention more than those farther away, we categorized target-present trials according to distractor proximity: close (adjacent to the target) or far (more than one position away). We then conducted a repeated-measures ANOVA on reaction times (RTs) from Experiments 1 A and 1B, with distractor type (real face vs. illusory face) and distractor proximity (close vs. far) as within-participant factors. The analyses revealed no significant main effects or interaction in either experiment (all ps > 0.12).

3 These presentation orders were designed to ensure that illusory face targets appeared in all possible positions (that is, in the first, second, or third block). When not presented in the first block, they were preceded once by the block with real faces as the target and once by the block with butterflies as the target.
