# Supplementary material for: Attentional capture by real and illusory faces: a failure to replicate
Source: Psychol Res. 2025 Nov 28;89(6):181. doi: 10.1007/s00426-025-02211-3 (PMC12662932; doi:10.1007/s00426-025-02211-3)
Supplement: Supplementary file 1 — Supplementary Material 1 (DOCX 3.49 MB) [file 426_2025_2211_MOESM1_ESM.docx]

**Supplementary analyses**

To assess differences in image characteristics among the various stimulus categories, we computed and analysed two distinct image descriptors. The first is the GIST descriptor, a fixed-length global image descriptor designed to capture the spatial layout and coarse structure—“the gist”—of a scene in a compact vector (Oliva & Torralba, 2001). The second is the Histogram of Oriented Gradients (HOG) descriptor, which captures local shape and appearance information by encoding the distribution of intensity gradients or edge directions within localized regions of an image. The underlying assumption is that the local image appearance and shape can be effectively characterized by the distribution of local intensity gradients, even without precise knowledge of their spatial arrangement.

*GIST descriptor.* To directly assess the visual similarity/dissimilarity between the target stimuli (real faces, illusory faces, butterflies) and the filler stimuli, we performed an image-level analysis using the GIST descriptor (Oliva & Torralba, 2001), as recently applied by Collyer et al. (2024). GIST descriptors were computed for each stimulus and arranged as feature vectors. Pairwise similarity was quantified using the Pearson correlation coefficient across descriptor dimensions and subsequently transformed into dissimilarities (1 – r), producing a representational dissimilarity matrix (RDM) that encodes the relative distances between images in terms of their global scene statistics (Figure 1).

Two ANOVAs were conducted on these dissimilarity values, followed by appropriate post-hoc pairwise comparisons (Bonferroni-corrected). The first ANOVA compared within-category values to assess which categories were more internally variable. This analysis showed that real faces were significantly more homogeneous (mean dissimilarity = 0.758, SD = 0.201, F = 13.70, *p* < .001, η²p = .034 ) than both standard objects (mean dissimilarity = 1.007, SD = .220; *t* = -5.941, *d* = - 1.137, *p* = < .001) and illusory faces (mean dissimilarity = 1.001, SD = .235; *t* = -4.152, *d* = - 1.1107, *p* = < .001), while butterflies showed intermediate values (mean dissimilarity = 0.900, SD = 0.182).

The second ANOVA compared the dissimilarity between each target category and fillers (standard objects), addressing whether any target was disproportionately similar to fillers. This analysis yielded no significant effects (F = 2.826, *p* = .060, η²p = .005). Thus, illusory faces do not seem to overlap more with fillers than the other categories (see Table 1).

To summarize, the results of the analyses revealed within-category differences for all stimulus categories, except for real faces, which exhibited greater overall homogeneity. Importantly, the differences between each target category (i.e., real faces, illusory faces, and butterflies) and the filler stimuli (standard objects) were comparable. Therefore, there was no indication of disproportionate similarity between illusory faces and fillers (that is, filler stimuli do not seem to disproportionately bias the visual similarity structure at the level of low-level image statistics).


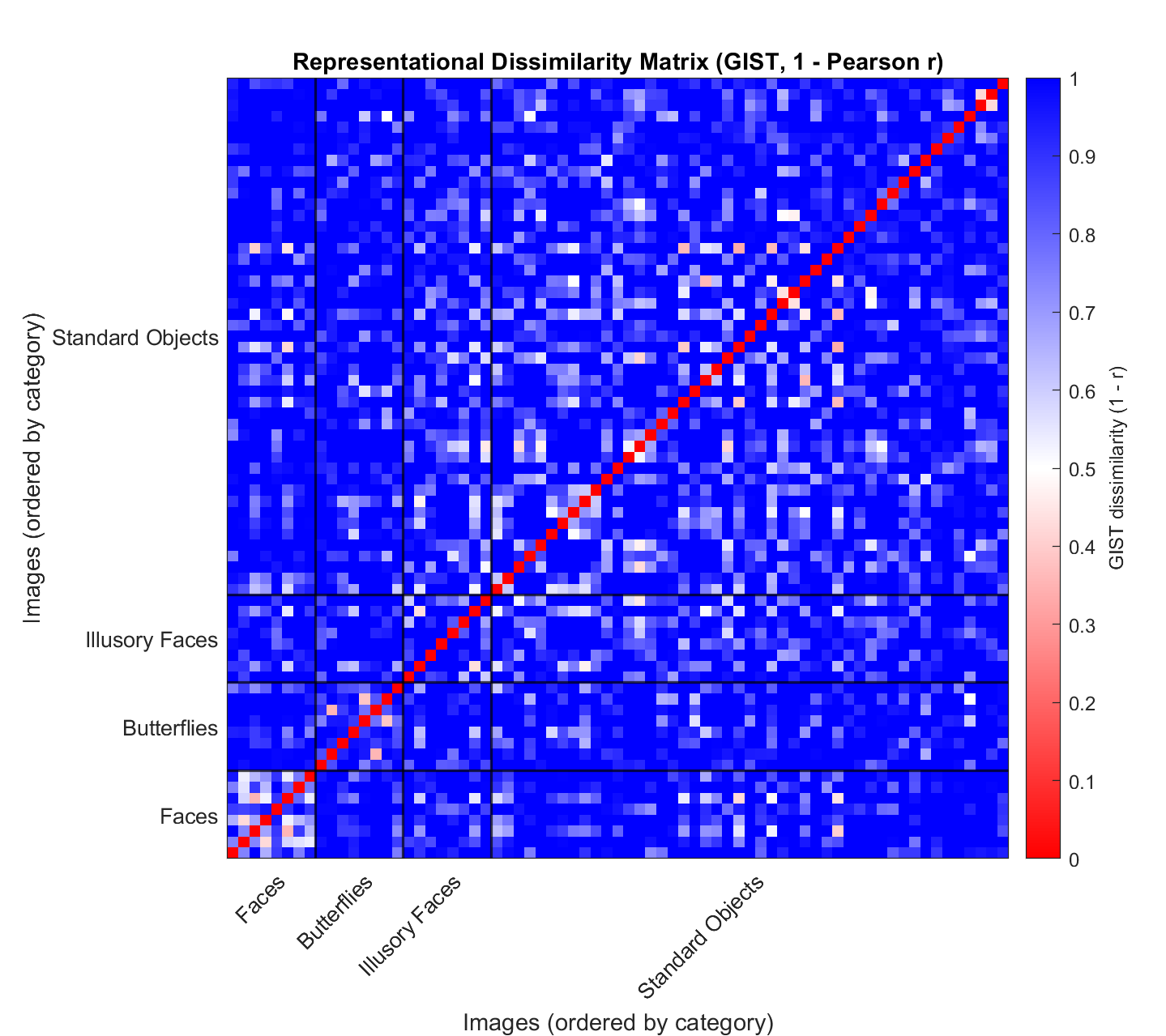


Figure 1. Representational Dissimilarity Matrix (RDM) based on GIST descriptors. Each cell represents the dissimilarity between two images, computed as 1−*r*, where *r* is the Pearson correlation between their GIST feature vectors. The resulting values range theoretically from 0 (perfectly correlated) to 2 (perfectly anticorrelated). For visualization purposes, dissimilarities were rescaled and clipped to the interval [0,1], so that higher values correspond to greater dissimilarity and lower values to greater similarity. Images are ordered by category (Faces, Butterflies, Illusory faces, Standard Objects), and black grid lines indicate category boundaries.

|  | |  | | Mean | | SD | |  |
| --- | --- | --- | --- | --- | --- | --- | --- | --- |
| Butterflies vs Standard Objects |  |  |  | 1.036 |  | 0.171 |  |  |
| Faces vs Standard Objects |  |  |  | 1.034 |  | 0.187 |  |  |
| Illusory Faces vs Standard Objects |  |  |  | 1.006 |  | 0.219 |  |  |

Table 1. Mean dissimilarity values (1 – *r*) between target categories (butterflies, faces, illusory faces) and fillers (Standard objects).

### *HOG descriptor.* To complement the global image analysis provided by the GIST descriptor, we extracted Histogram of Oriented Gradients (HOG) features (Dalal & Triggs, 2005; Felzenszwalb et al., 2010) to capture fine-grained local shape and edge information within each image. All stimuli were resized to 128 × 128 pixels and analysed using a custom implementation of the HOG algorithm. Local image gradients were computed using horizontal and vertical derivative filters, and for each 8 × 8-pixel cell, a histogram of gradient orientations was constructed with nine unsigned orientation bins (0–180°). These histograms were normalized within overlapping 2 × 2 cell blocks using L2-Hys normalization to ensure robustness to changes in contrast and illumination. The concatenation of all normalized block histograms yielded a high-dimensional feature vector representing each image’s local structural composition. The resulting feature matrix was mean-centered and submitted to Principal Component Analysis (PCA) based on singular value decomposition (Jolliffe & Cadima, 2016). The PCA scores provided a low-dimensional embedding of the stimuli, which was analysed using a series of one-way ANOVAs conducted on the first ten principal components (PC1–PC10), with image category (*Faces, Illusory Faces, Butterflies, Standard Objects*) as the between-subjects factor.

Table 2 summarizes the results of the analysis. Most components (PC1, PC4–PC7, PC9–PC10) showed no significant main effect of category (all *p* > .05), with significant effects observed only for PC2, PC3, and PC8. Specifically, for PC2 (*F*(3,59) = 2.92, *p* = .041, η² = .13), Bonferroni-corrected post hoc tests revealed that butterfly images scored significantly higher than faces (*p* = .035), while all other pairwise comparisons were non-significant (*p* > .30). For PC3 (*F*(3,59) = 9.29, *p* < .001, η² = .32), real faces scored significantly higher than all other categories (all *p_bonf_* ≤ .001), confirming that this component captures local structural features characteristic of facial configurations. Finally, for PC8 (*F*(3,59) = 3.82, *p* = .014, η² = .16), post hoc comparisons indicated a significant difference between Faces and Butterflies (*p_bonf_*= .046), while other contrasts were non-significant.
Overall, these findings suggest that only a subset of HOG-derived components—particularly PC3, and to a lesser extent PC2 and PC8—reliably differentiate between categories, primarily distinguishing faces from non-face stimuli based on variations in local edge and texture organization.

| **Component** | **F** | **p-value** | **η² (partial)** |
| --- | --- | --- | --- |
| PC1 | 0.90 | .447 | .04 |
| **PC2** | **2.92** | **.041** | **.13** |
| **PC3** | **9.29** | **< .001** | **.32** |
| PC4 | 2.09 | .112 | .10 |
| PC5 | 0.28 | .841 | .01 |
| PC6 | 2.27 | .090 | .10 |
| PC7 | 1.51 | .223 | .07 |
| **PC8** | **3.82** | **.014** | **.16** |
| PC9 | 0.95 | .421 | .05 |
| PC10 | 0.82 | .490 | .04 |

### Table 2. One-way ANOVAs on HOG PCA components (factor: Image Category)

**References**

Oliva, A., Torralba, A. (2001). Modeling the Shape of the Scene: A Holistic Representation of the Spatial Envelope. *International Journal of Computer Vision,* 42, 145–175. Doi: 10.1023/A:1011139631724

Dalal, N., & Triggs, B. (2005). *Histograms of oriented gradients for human detection.* In Proceedings of the IEEE Computer Society Conference on Computer Vision and Pattern Recognition (CVPR) (Vol. 1, pp. 886–893). IEEE.

Felzenszwalb, P. F., Girshick, R. B., McAllester, D., & Ramanan, D. (2010). *Object detection with discriminatively trained part-based models.* IEEE Transactions on Pattern Analysis and Machine Intelligence, 32(9), 1627–1645.

Jolliffe, I. T., & Cadima, J. (2016). *Principal component analysis: a review and recent developments.* Philosophical Transactions of the Royal Society A, 374(2065), 20150202.
